# Comparison of estrogens and estrogen metabolites in human breast tissue and urine

**DOI:** 10.1186/1477-7827-8-93

**Published:** 2010-08-02

**Authors:** Emanuela Taioli, Annie Im, Xia Xu, Timothy D Veenstra, Gretchen Ahrendt, Seymour Garte

**Affiliations:** 1 SUNY Downstate Medical Center, Brooklyn, New York 11203, USA; 2 Graduate School of Public Health, University of Pittsburgh, Pittsburgh, Pennsylvania 15232, USA; 3 Department of Surgery, Division of Surgical Oncology, University of Pittsburgh, Pittsburgh, Pennsylvania 15232, USA; 4 Laboratory of Proteomics and Analytical Technologies, Advanced Technology Program, SAIC- Frederick, Inc., NCI-Frederick, Frederick, Maryland 21702, USA

## Abstract

**Background:**

An important aspect of the link between estrogen and breast cancer is whether urinary estrogen levels are representative of the intra-tissue levels of bioavailable estrogens.

**Methods:**

This study compares 15 estrogen and estrogen metabolite levels in breast tissue and urine of 9 women with primary breast cancer using a quantitative liquid chromatography-mass spectrometry method.

**Results:**

The average levels of estrogens (estrone, 17 beta-estradiol) were significantly higher in breast tissue than in urine. Both the 2 and the 16-hydroxylation pathways were less represented in breast tissue than urine; no components of the 4-hydroxypathway were detected in breast tissue, while 4-hydroxyestrone was measured in urine. However, the 2/16 ratio was similar in urine and breast tissue. Women carrying the variant CYP1B1 genotype (Leu/Val and Val/Val) showed significantly lower overall estrogen metabolite, estrogen, and 16-hydroxylation pathway levels in breast tissue in comparison to women carrying the wild type genotype. No effect of the CYP1B1 polymorphism was observed in urinary metabolites.

**Conclusions:**

The urinary 2/16 ratio seems a good approximation of the ratio observed in breast tissue. Metabolic genes may have an important role in the estrogen metabolism locally in tissues where the gene is expressed, a role that is not readily observable when urinary measurements are performed.

## Background

A woman's lifetime exposure to estrogens has been widely accepted as a risk factor for breast cancer [1-3]. Proposed mechanisms for the association include the ability of estrogens to induce breast cell proliferation, stimulate cell division [4,5], and increase oxidative damage with a direct genotoxic effect [6].

Estrogen metabolites have also been extensively studied as breast cancer risk factors. This process involves an oxidative pathway, beginning with the conversion of estradiol to estrone, and continues through hydroxylation at sites C2, C4 or C-16 [6]. Both 2-OHE_1 _and 16α-OHE_1 _are estrogen-like compounds, however, the 16α-OHE_1 _metabolite is a potent estrogenic molecule that activates the ER and induces proliferation of cultured breast cancer cells by 40%, whereas the 2-OHE_1 _metabolite has very little estrogen receptor binding affinity (< 0.1% compared to estradiol) [7], and has been shown to decrease cell proliferation by 20 to 30% in cultured breast cancer cell lines [8,9]. Components of the 4-hydroxylation pathway also have been shown to be genotoxic [7,10]; the 4OHE_1 _metabolite is thought to be strongly estrogenic and potentially carcinogenic, yet quantitatively its production is generally less than 15% of the production of 2OHE_1 _[7].

Although limited research has been conducted in humans on the effects on estrogen metabolites of metabolic gene polymorphisms involved in estrogen metabolism, a common CYP1B1 polymorphism (Val432Leu) has been shown to influence the 2/16 ratio in healthy women, possibly by catalyzing the formation of the 2 hydroxyl group [11].

Several epidemiological studies have investigated the association between 2-OHE_1 _and 16α-OHE_1 _levels and breast cancer [12-23]. While the prevailing hypothesis suggests that a higher 2-OHE_1 _to 16α-OHE_1 _ratio would predict a lower breast cancer risk, the results have been inconsistent. Methodological reasons such as the variability of estrogen levels and metabolism across the menstrual cycle, the effect of both genetic and environmental factors on estrogen production and metabolism, and the use of urine or serum measurements as surrogate markers of breast tissue estrogen metabolism, may be the basis of the varying results provided by the different studies.

An important aspect of the link between estrogen levels and breast cancer risk that has not been addressed is whether urinary and plasma estrogen metabolite levels are representative of the intra-tissue levels of bioavailable estrogens. Human breast tissue estrogens levels have been reported to be much higher than their respective plasma values, and several key enzymes involved in steroid metabolism are specifically expressed in both normal and malignant human breast tissues [24-27].

The present study compares estrogen and estrogen metabolite levels in breast tissue and urine of women who were diagnosed with primary breast cancer using a quantitative liquid chromatography-mass spectrometry method. The role of one of the metabolic genes involved in estrogen metabolism, CYP1B1, is also studied.

## Methods

### Breast tissue and urine sample collection

Women who were diagnosed with primary, incident breast cancer pathologically confirmed at Magee-Women's Hospital at the University of Pittsburgh during the period 2006-2008 were asked to answer questions from a standardized survey including data on family history of cancer, smoking history, and alcohol use. Patients' height and weight were obtained to calculate their BMI. Clinical annotations were extracted from electronic medical records.

Morning spot urine samples were obtained at the time of visit, pre-surgery/pre-therapy, preserved by addition of 400 mg ascorbic acid and frozen at -20°C after collection. None of the subjects had received estrogen-containing treatment for at least 3 months prior to urine sample collection. Informed consent was obtained from each patient prior to survey administration, sample collection and linkage to the breast tissue specimen collected at surgery. Study procedures were approved by the University of Pittsburgh Medical Center Institutional Board.

Breast tissues samples were snap frozen at the time of surgery, and stored in liquid nitrogen. To reduce variability due to personal characteristics, only samples from white women who declared themselves to be never smokers were considered eligible. Among the eligible women, a stratified random sample was extracted according to CYP1B1 genotype. The final sample presented in this study consisted of 9 women.

### Sample preparations

Sample preparations for urine samples and breast tissues were conducted as previously described [28,29]. Unconjugated estrogens were extracted from human samples using dichloromethane. The SI-EM internal standards were incorporated at the beginning stages of sample preparations to compensate for the potential loss of any EM during sample manipulations. Extracted EM and SI-EM were dansylated prior to liquid spectrometry analyses.

### Laboratory methods

Fifteen estrogen metabolites (EM) [estrone (E_1_), estradiol (E_2_), estriol (E_3_), 16-epiestriol (16-epiE_3_), 17-epiestriol (17-epiE_3_), 16-ketoestradiol (16-ketoE_2_), 16α-hydroxyestrone (16α-OHE_1_), 2-methoxyestrone (2-MeOE_1_), 4-methoxyestrone (4-MeOE_1_), 2-hydroxyestrone-3-methyl ether (3-MeOE_1_), 2-methoxyestradiol (2-MeOE_2_), 4-methoxyestradiol (4-MeOE_2_), 2-hydroxyestrone (2-OHE_1_), 4-hydroxyestrone (4-OHE_1_), and 2-hydroxyestradiol (2-OHE_2_)] were obtained from Steraloids, Inc. (Newport, RI). Stable isotope labeled estrogens (SI-EM) estradiol-13,14,15,16,17,18-^13^C_6 _(^13^C_6_-E_2_) and estrone-13,14,15,16,17,18-^13^C_6 _(^13^C_6_-E_1_) were purchased from Cambridge Isotope Laboratories, Inc. (Andover, MA). The SI-EM estriol-2,4,17-*d*_3 _(d_3_-E_3_), 2-hydroxyestradiol-1,4,16,16,17-*d*_5 _(d_5_-2-OHE_2_), and 2-methoxyestradiol-1,4,16,16,17-*d*_5 _(d_5_-2-MeOE_2_), were obtained from C/D/N Isotopes, Inc. (Pointe-Claire, Quebec, Canada), and 16-epiestriol-2,4,16-*d*_3 _(d_3_-16-epiE_3_) was purchased from Medical Isotopes, Inc. (Pelham, NH). These analytical standards have reported chemical and isotopic purity ≥98%. Dichloromethane and methanol were obtained from EMD Merck KGaA (Darmstadt, Germany). Sodium hydroxide and sodium acetate were purchased from Fisher Scientific (Fair Lawn, NJ). Glacial acetic acid, formic acid, sodium bicarbonate, L-ascorbic acid were obtained from Sigma Chemical Co. (St. Louis, MO). Dansyl chloride and acetone were purchased from Aldrich Chemical Co. (Milwaukee, WI). All chemicals and solvents utilized in these experiments were high purity or HPLC grade.

### Liquid Chromatography-Tandem Mass Spectrometry (LC-MS^2^)

Capillary LC-MS^2 ^analysis was conducted using an Agilent 1200 series nanoflow LC system (Agilent Technologies, Palo Alto, CA) coupled to a TSQ™ Quantum Ultra triple quadrupole mass spectrometer (Thermo Electron, San Jose, CA). The LC separation was carried out using a 150 mm long × 300 μm i.d. column packed with 4 μm Synergi Hydro-RP particles (Phenomenex, Torrance, CA) maintained at 40°C. Eight microliters of each sample was injected onto the column. The mobile phase, operating at a flow rate of 4 μL/min, consisted of methanol as solvent A and 0.1% (v/v) formic acid in water as solvent B. A linear gradient from 72-85% solvent B in 75 min. was used to resolve the EM and SI-EM. Mass spectrometry conditions were as follows: source: ESI; ion polarity: positive; spray voltage: 3500 V; sheath and auxiliary gas: nitrogen; sheath gas pressure: 7 arbitrary units; ion transfer capillary temperature, 270°C; scan type: selected reaction monitoring (SRM); collision gas: argon; collision gas pressure: 1.5 mTorr; scan width: 0.7 u; scan time: 0.50 s; Q1 peak width: 0.70 u full-width half-maximum (FWHM); Q3 peak width: 0.70 u FWHM. The optimized SRM conditions for the protonated molecules [MH]^+ ^of EM-Dansyl and SI-EM-Dansyl were similar to those previously described [28,29].

### Quantitation of estrogen metabolites

Xcalibur™ Quan Browser (Thermo Electron) was used to quantitate the urine and tissue EM as previously described [28,29]. Calibration curves for each EM were constructed by plotting EM-Dansyl/SI-EM-Dansyl peak area ratios obtained from calibration standards versus amounts of EM injected on column and fitting these data using linear regression with 1/*X *weighting. The amounts of EM in sample were interpolated using this linear function. Based on their similarity of structures and retention times, ^13^C_6_-E_2 _was used as the internal standard for E_2_; ^13^C_6_-E_1 _for E_1_; d_3_-E_3 _for E_3_, 16-ketoE_2_, and 16α-OHE_1_; d_3_-16-epiE_3 _for 16-epiE_3 _and 17-epiE_3_; d_5_-2-MeOE_2 _for 2-MeOE_2_, 4-MeOE_2_, 2-MeOE_1_, 4-MeOE_1_, and 3-MeOE_1_; d_5_-2-OHE_2 _for 2-OHE_2_, 2-OHE_1_, and 4-OHE_1, _respectively. All samples were run in triplicate; in addition, approximately 1/3 of the samples analyzed were used for quality control or as calibration standards.

Metabolites recovery rate: the recovery rates in urine were measured using quality control samples containing 0.12, 0.96, and 6.4 ng/mL of each estrogen metabolite; the percent recovery ranged from 98-106%, 96-103%, and 97-107%, respectively. The intra-batch precision, as estimated from four replicate urine analyses at each concentration level, was 2-5%, 1-5%, and 1-3% RSD for the 0.12, 0.96, and 6.4 ng/mL control urine samples, respectively. The lower limit of quantitation for each estrogen is 0.02 ng/0.5 mL urine sample (2 pg on column). The limit of detection is 250 fg of each estrogen metabolite on-column.

Assay reliability: The inter-assay variation coefficients of the analytical method in urine, measured using the lowest quality control standard containing 0.12 pg/mL of each estrogen metabolite, range from a low of 3.8% for 16-epiE_3 _to a high of 12.1% for 16αOHE_1_. The intra-assay variation ranges from a low of 2.1% for 4MeOE_1 _to a high of 5.1% for 16αketoE_2_. In five serial tissue sections cut from a lymph node infiltrated with metastatic breast cancer [29], the coefficients of variation ranged from a low of 2.4% for E_1 _to a high of 12.4% for 2-OHE_2_.

### CYP1B1 genotype

Genomic DNA was extracted from buffy coats and analyzed for the presence of the G to C transversion at codon 432 of the CYP1B1 gene by a PCR-based Restriction Fragment Length Polymorphism (RFLP) assay. PCR amplification of a 650 bp fragment of the CYP1B1 gene, including part of exon 3 that contains the polymorphism was carried out using forward primer: TCACTTGCTTTTCTCTCTCC and reverse primer: AATTTCAGCTTGCCTCCTG. Experimental details are described elsewhere [11]. Restriction digestion of the DNA fragment was carried out using Eco57I restriction enzyme. The product of the restriction digest was mixed with 10 μl of loading dye and verified on a 3% agarose gel (with Ethidium bromide) electrophoresis in a 1× Tris-Borate-EDTA buffer at 200 V for 60 min. The presence of a G at position 1294 (CYP1B1-codon 432) generated a unique 650 bp fragments, while the 650 bp fragment was divided into unique 340 bp and 310 bp fragments when position 1294 contains a C. The gels were visualized by UV light and the RFLP gel electrophoresis products were read by two independent persons.

### Statistical analysis

Abundances of individual EM were summed to obtain the 2-, 4-, and 16α-estrogen hydroxylation pathway levels. The 2-hydroxylation pathway includes the 2-hydroxyestrone, 2-hydroxyestradiol, 2-methoxyestrone, 2-methoxyestradiol, and 2-hydroxyestrone-3-methyl-ether measures. The 4-hydroxylation pathway includes 4-hydroxyestrone, 4-methoxyestrone, and 4-methoxyestradiol, and the 16α-hydroxylation pathway includes 16α-hydroxyestrone, estriol, 17-epiestriol, 16-epiestriol, and 16-ketoestradiol. When comparisons between urine and tissue are presented, the ratio of individual metabolites to the entire levels of all estrogen recovered from each compartment is calculated and expressed as percent distribution.

Data are presented as mean ± SD; stratified data according to CYP1B1 genotype are presented as mean ± SE. The Kruskal-Wallis rank test was used to compare overall averages from breast tissue and urine, and EM across categories of CYP1B1 polymorphism. Comparisons between tissue and urine percent EM from each subject was performed by paired t-test. All the analyses were conducted using SAS v9.2 (Cary, NC).

## Results

The general and clinical characteristics of breast cancer cases included in this study are reported in Table 1. Because of the inclusion criteria set up *a priori*, all the cases were white non-smokers. Average age was 56.2 ± 10.2 years, average BMI was 31.5 ± 9.8 kg/m^2^; two women claimed to be occasional drinkers of alcoholic beverages and seven reported to be in natural post-menopause at the time of cancer diagnosis

**Table 1:** Description of breast cancer cases included in the study

ID	HISTOLOGY	Age at diagnosis (years)	**Race**	Smoking	Drinking	Menopausal status	BMI (Kg/m^2^)	CYP1B1 (V432L) *
5	Intra Ductal Carcinoma	68	White	Never	Unknown	Post	51.16	Wild type
8	Intra Ductal Carcinoma	45	White	Never	No	Pre	38.39	Wild type
9	Intra Ductal and Lobular Carcinoma	51	White	Never	No	Post	26.63	Wild type
1	Intra Ductal Carcinoma	44	White	Never	Yes	Pre	22.49	Heterozygous
2	Intra Ductal Carcinoma	56	White	Never	No	Post	37.12	Heterozygous
6	Intra Ductal Carcinoma	69	White	Never	No	Post	35.51	Heterozygous
3	Intra Ductal Carcinoma	60	White	Never	No	Post	21.45	Homozygous variant
4	Intra Ductal Carcinoma	67	White	Never	Yes	Post	27.36	Homozygous variant
7	Metaplastic carcinoma	46	White	Never	No	post	23.51	Homozygous variant

*SNP rs1056836

Among the various EM measured in breast tissue and urine (Table 2), percent estrogen levels (estrone, 17β-estradiol) were significantly higher in breast tissue on average than in urine. Average relative products of the 16-hydroxylation pathway were significantly lower in breast tissue than in urine, and in this pathway 17-epiestriol was undetectable in breast tissue. Several non-significant differences between overall tissue and urine values were observed: the 2-hydroxylation pathway was less represented in breast tissue than urine, mostly because of the complete absence of 2-hydroxyestrone and 2-hydroxy-17β-estradiol in breast tissue as compared to urine. Similarly, no components of the 4-hydroxypathway were detected in breast tissue, while 4-hydroxyestrone was measured in urine. The ratio between the 2 and the 16 pathways was slightly higher in breast tissue than in urine, although the difference was not statistically significant. The sum of all EM levels measured in tissue, but not in urine, significantly correlated with BMI. No correlation with age was observed for any metabolite.

**Table 2:** Mean values of relative estrogen metabolites measured in breast tissue and corresponding urines

ESTROGEN METABOLITES (% of total estrogen)	BREAST TISSUE Mean ± SD	URINE Mean ± SD	P value (Kruskal-Wallis test)
**Estrogens **(estrone, 17β-estradiol)	53.4 ± 19.0	20.5 ± 18.4	0.02
2-Hydroxylation Pathway (*)	15.6 ± 8.7	21.4 ± 10.3	0.4
16-Hydroxylation Pathway (**)	30.9 ± 14.7	55.4 ± 17.8	0.02
**4-hydroxyestrone (^#^)**	**--**	2.7 ± 2.0	N/A
**2/16 ratio**	0.61 ± 0.5	0.47 ± 0.34	0.45
	**pg/g wet tissue**	**pg/mL**	
**TOTAL**	**348.1 ± 188.8**	**337.4 ± 310.7**	0.45

*2-hydroxyestrone-3-methyl ether was not detected in breast tissue or urine

**16-keto-17β-estradiol, 16-epiestriol were not detected in breast tissue or urine

**^#^**4-methoxyestrone, 4-methoxy-17β-estradiol were not detected in breast tissue or urine

**Figure 1:**
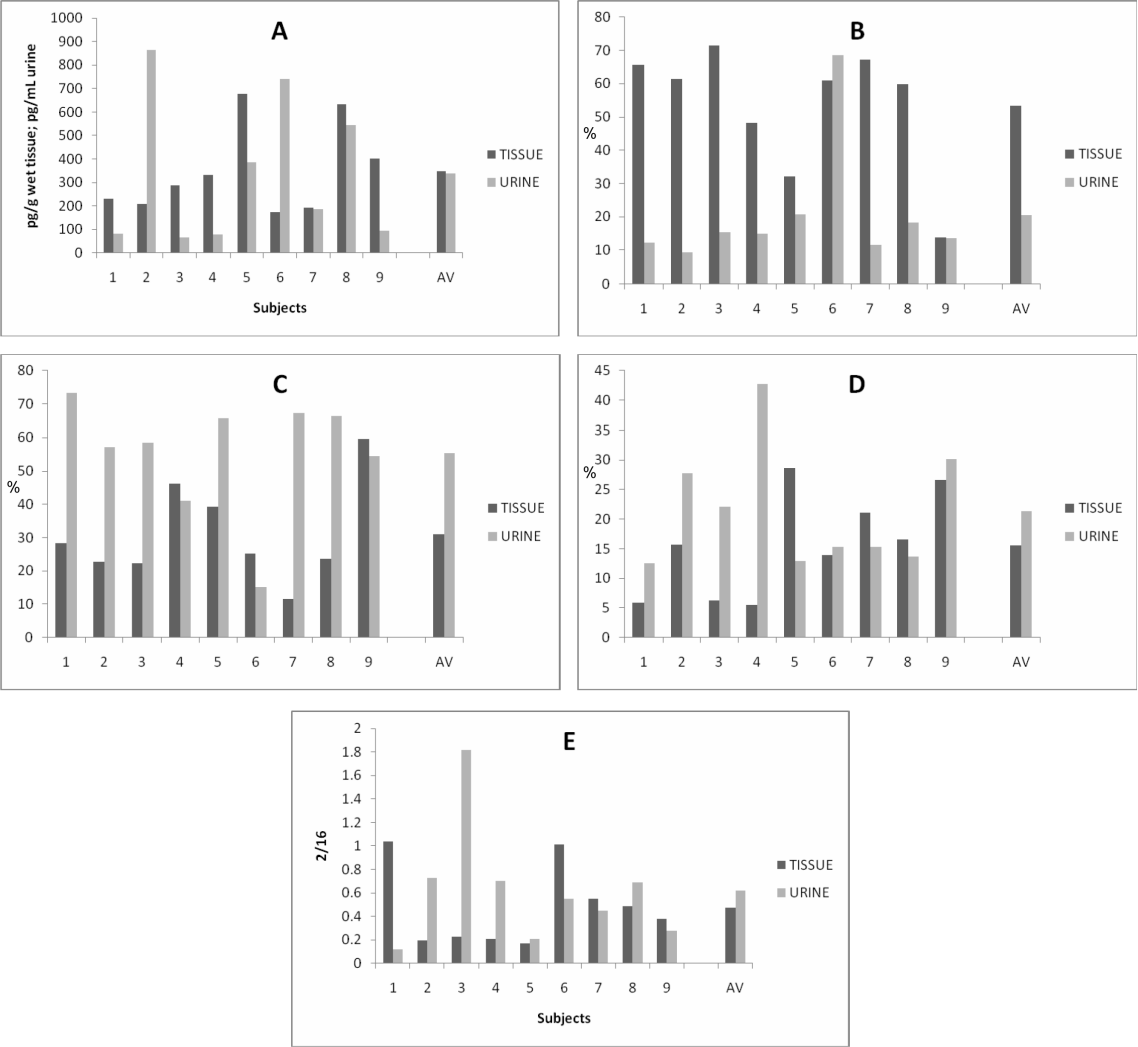
**Individual comparison of EM measured in breast tissue and corresponding urine**. **A**) Total metabolites, p value (paired t-test): 0.9. **B**) Percent total estrogens (estrone, 17β-estradiol), p value (paired t-test): 0.005. **C**) Percent 16-hydroxylation Pathway, p value (paired t-test): 0.3. **D**) percent 2-hydroxylation Pathway, p value (paired t-test): 0.02. **E**) 2/16 ratio), p value (paired t-test): 0.56. AV = average.

The individual correspondence between relative values measured in urine and breast tissue is shown in Figure 1. There was a statistically significant difference in individual relative levels of estrogen, and products of the 16-hydroxylation between breast tissue and corresponding urine obtained from the same subject; the general pattern of the 2/16 ratio showed a correspondence between urine and tissue; urine measurements showed in general a much larger variability than tissue measurements.

When data were stratified according to the CYP1B1 I462V polymorphism (Table 3), women carrying the variant genotype (Leu/Val and Val/Val) showed significantly lower average values of EM, estrogens, and 16-hydroxylation pathway levels in breast tissue in comparison to women carrying the wild type genotype. No effect of the CYP1B1 polymorphism was observed in urinary EM. The 2/16 ratio, either measured in urine or in breast, was not significantly affected by the presence of the CYP1B1 polymorphism.

**Table 3:** Association between CYP1B1 Leu/Val polymorphism and EM levels in breast tissue and urine

BREAST TISSUE pg/g wet tissue	CYP1B1 Leu/Leu Mean (SE)	CYP1B1 Leu/Val+ Val/Val Mean (SE)	p-value (Kruskal-Wallis test)
Estrogens^#^	373.7 (46.2)	117 (28)	0.02
2-hydroxylation Pathway	54.7 (22.9)	43.9 (8.3)	0.79
16-hydroxylation Pathway	141.8 (29.7)	76.1 (13.2)	0.07
Total*	570.5 (85.7)	237.0 (25.2)	0.02
2/16 ratio	0.39 (0.15)	0.73 (0.24)	0.31
**URINE pg/mL**			
Estrogens	37.4 (11.7)	124.3 (81.2)	0.79
2-hydroxylation Pathway	73.3 (39.6)	56.1 (19.2)	0.6
16-hydroxylation Pathway	216.6 (81.5)	151.5 (84)	0.3
Total	341.8 (132.3)	335.5 (149.1)	0.6
2/16 ratio	0.34 (0.09)	0.54 (0.16)	0.43

*P value test for trend = 0.05

^#^P value test for trend 0.06

## Discussion

This study compared the levels of unconjugated estrogen metabolites in breast tissue and urine specimens from the same individuals. To measure the complete spectrum of EM, a highly sensitive liquid chromatography-tandem mass spectrometry method was used. Despite that fact that the sum of the EM levels was similar between breast tissue and urine, the levels of many of the individual components were quite different between these two tissue sources. The sum of the estrogen levels (estrone, 17β-estradiol) was higher in breast tissue than urine, while the metabolites of the 2 and the 16-hydroxylation pathways were lower. This result is consistent with previous reports [7] suggesting rapid conjugation of the products of 2 hydroxylation pathway in tissues, followed by excretion in the urine. For the catechol estrogen pathway, only the methoxylated metabolites were detected in breast tissue, again suggesting rapid methoxylation and excretion of tissue metabolites. The metabolites of the 4 and 16 hydroxylation pathways were also present in higher concentration in urine than in breast tissue. It is clear that urinary and breast tissue concentrations of specific estrogen metabolites reflect the specific metabolic profile in tissue as well as the degree of urinary excretion of each metabolite. It is possible that some of the metabolites detected in urine originate from organs other than breast [30].

Two other studies have been published on EM measurement in breast tissue from human subjects [31,32] with the purpose of assessing differences between breast cancer patients and healthy controls, but no individual comparisons between breast tissue and urine has been performed so far. A comparison between the EM values measured in our breast tissue samples with those reported by the two publications is not feasible, since different laboratory methods were used for detection. Both of these groups used high performance liquid chromatography with electrochemical detection to assess 11 conjugated estrogen derivatives. The present study used LC-MS^2 ^to measure 15 unconjugated products [33].

Aside from the detailed metabolic activities that might lead to differences in breast tissue and urinary concentrations of specific estrogen metabolites, it is of interest to compare the metabolite levels in the two tissue sources, to determine the value of urinary measurements as a surrogate biomarker of estrogen metabolite levels in breast. The collection of urine for molecular epidemiologic studies represents a very simple approach which is usually well accepted by the study participants; specifically, estrogen metabolites in urines have been used as markers of susceptibility to breast cancer in healthy women at high risk for breast cancer [34].

Our results suggest, in a small sample of women, that the sum of the estrogen metabolite levels in urine do reflect that formed in the target organ human breast tissue, while this is much less true for most individual metabolites. Among the various products measured, our study showed that the 2/16 ratio was quite similar between breast tissue and urine, thus supporting the use of the ratio in urine as a surrogate tissue; it can be therefore assumed that the urinary ratio measured in a woman has a fair corresponds with the ratio that would be measured in her breast tissue, if a biopsy could be performed.

The production of estrone and 17β-estradiol has been described to be under control of the CYP19 gene [35], while CYP1B1 encodes for an enzyme that catalyzes the formation of 2- and 4-hydroxyl estrone [7]. When the presence of a common CYP1B1 polymorphism was examined as a possible modifier of the EM levels in breast and urine, we observed that the variant CYP1B1 allele was associated with lower total metabolites and estrogen levels in breast tissue but not in urine. CYP1B1 is expressed in the breast as well as in kidney, prostate, uterus, ovary and placenta from human subjects [7,36]. These observations suggest that metabolic genes may have an important role in the estrogen metabolism locally in tissues where the gene is expressed, a role that is not readily observable when urinary measurements are performed. The CYP1B1 genotype had no significant effect on the 2/16 ratio in either breast tissue or urine, thus highlighting the use of the 2/16 ratio as an independent biomarker of breast cancer risk.

A complete profile of genotypes of genes regulating estrogen metabolism in breast tissue would be useful in further understanding individual differences in estrogen metabolism in both the healthy breast and in breast cancer. Another aspect that still needs to be studied is the validity of these findings in other ethnic groups. In fact, the present study included a small sample of white women only.

## Conclusions

The urinary 2/16 ratio is a good approximation of the ratio observed in breast tissue. Metabolic genes may have an important role in the estrogen metabolism locally in tissues where the gene is expressed, a role that is not readily observable when urinary measurements are performed.

## Competing interests

The authors declare that they have no competing interests.

## Authors' contributions

ET designed the study, SG performed the genotype, GA enrolled the patients, XX and TV performed the estrogen measurements. AI reviewed the clinical charts and prepared the data base for analysis. All authors contributed to manuscript writing. All authors read and approved the final manuscript. 

## References

[B1] Bernstein L Epidemiology of Endocrine-Related Risk Factors for Breast Cancer J Mammary Gland Biol Neopl 2002 7 1 3 15 10.1023/A:1015714305420 12160084

[B2] Henderson BE Fiegelson HS Hormonal carcinogenesis Carcinogenesis 2000 21 427 433 10.1093/carcin/21.3.427 10688862

[B3] Key TJ Appleby PN Barnes I Reeves G Endogenous Hormones and Breast Cancer Collaborative Group. Endogenous sex hormones and breast cancer in postmenopausal women: reanalysis of nine prospective studies JNCI 2002 94 606 616 11959894 10.1093/jnci/94.8.606

[B4] Preston-Martin S Pike MC Ross RK Jones PA Henderson BE Increased cell division as a cause of human cancer Cancer Res 1990 50 7415 7421 2174724

[B5] Feigelson HS Henderson BE Estrogens and breast cancer Carcinogenesis 1996 17 2279 2284 10.1093/carcin/17.11.2279 8968038

[B6] Yager JD Davidson NE Estrogen carcinogenesis in breast cancer N Engl J Med 2006 354 270 282 10.1056/NEJMra050776 16421368

[B7] Zhu BT Conney AH Is 2-methoxyestradiol an endogenous estrogen metabolite that inhibits mammary carcinogenesis? Cancer Res 1998 58 2269 2277 9622057

[B8] Lippert C Seeger H Mueck AO The effect of endogenous estradiol metabolites on the proliferation of human breast cancer cells Life Sci 2003 72 877 883 10.1016/S0024-3205(02)02305-6 12493568

[B9] Seeger H Wallwiener D Kraemer E Mueck AO Comparison of possible carcinogenic estradiol metabolites: effects on proliferation, apoptosis and metastasis of human breast cancer cells Maturitas 2006 54 72 77 10.1016/j.maturitas.2005.08.010 16213115

[B10] Sepkovic DW Bradlow HL Estrogen hydroxylation--the good and the bad Ann N Y Acad Sci 2009 1155 57 67 10.1111/j.1749-6632.2008.03675.x 19250192

[B11] Paracchini V Pedotti P Raimondi S Garte S Bradlow HL Sepkovic DW Taioli E A common CYP1B1 polymorphism is associated with 2-OHE1/16-OHE1 urinary estrone ratio Clin Chem Lab Med 2005 43 7 702 706 10.1515/CCLM.2005.119 16207128

[B12] Schneider J Kinne D Fracchia A Pierce V Anderson KE Bradlow HL Fishman J Abnormal oxidative metabolism of estradiol in women with breast cancer Proc Natl Acad Sci 1982 79 3047 3051 10.1073/pnas.79.9.3047 6953448 PMC346346

[B13] Fishman J Schneider J Hershcope RJ Bradlow HL Increased estrogen-16α-hydroxylase activity in women with breast and endometrial cancer J Steroid Biochem 1984 20 1077 1081 10.1016/0022-4731(84)90021-9 6727352

[B14] Cauley J Zmuda J Danielson ME Ljung BM Bauer DC Cummings SR Kuller LH Estrogen metabolites and the risk of breast cancer in older women Epidemiology 2003 14 740 744 10.1097/01.ede.0000091607.77374.74 14569192

[B15] Fowke J Qi D Bradlow HL Shu XO Gao YT Cheng JR Jin F Zheng W Urinary estrogen metabolites and breast cancer: differential pattern of risk found with pre-versus post-treatment collection Steroids 2003 68 65 72 10.1016/S0039-128X(02)00116-2 12475724

[B16] Kabat G Chang CJ Sparano JA Sepkovic DW Hu XP Khalil A Rosenblatt R Bradlow HL Urinary estrogen metabolites and breast cancer: a case-control study Cancer Epidemiol Biomarkers Prev 1997 6 7 505 509 9232337

[B17] Kabat G O'Leary ES Gammon MD Sepkovic DW Teitelbaum SL Britton JA Terry MB Neugut AI Bradlow HL Estrogen metabolism and breast cancer Epidemiology 2006 17 80 88 10.1097/01.ede.0000190543.40801.75 16357599

[B18] Meilahn E DeStavola B Allen D Fentiman I Bradlow HL Sepkovic DW Kuller LH Do urinary oestrogen metabolites predict breast cancer? Guernsey III cohort follow-up Br J Cancer 1998 78 9 1250 1255 9820189 10.1038/bjc.1998.663 PMC2063014

[B19] Modugno F Kip K Cochrane B Kuller L Klug TL Rohan TE Chlebowski RT Lasser N Stefanick ML Obesity, hormone therapy, estrogen metabolism and risk of postmenopausal breast cancer Int J Cancer 2006 118 5 1292 1301 10.1002/ijc.21487 16161054

[B20] Muti P Bradlow H Micheli A Krogh V Freudenheim JL Schünemann HJ Stanulla M Yang J Sepkovic DW Trevisan M Berrino F Estrogen metabolism and risk of breast cancer: A prospective study of the 2:16α-hydroxyestrone ratio in premenopausal and postmenopausal women Epidemiology 2000 11 635 640 10.1097/00001648-200011000-00004 11055622

[B21] Ursin G London S Stanczyk F Gentzschein E Paganini-Hill A Ross RK Pike MC Urinary 2-hydroxyestrone/16α-hydroxyestrone ratio and risk of breast cancer in postmenopausal women JNCI 1999 91 1067 1072 10379970 10.1093/jnci/91.12.1067

[B22] Wellejus A Olsen A Tjonneland A Thomsen BL Overvad K Loft S Urinary hydroxyestrogens and breast cancer risk among postmenopausal women: a prospective study Cancer Epidemiol Biomarkers Prev 2005 14 9 2137 2141 10.1158/1055-9965.EPI-04-0934 16172222

[B23] Ursin G London S Yang D Tseng CC Pike MC Bernstein L Stanczyk FZ Gentzschein E Urinary 2-hydroxyestrone/16α-hydroxyestrone ratio and family history of breast cancer in premenopausal women Breast Cancer Res Treat 2002 72 139 143 10.1023/A:1014896417653 12038704

[B24] van Landeghem AAJ Poortman J Nabuurs M Thijssen JHH Endogenous concentration and subcellular distribution of estrogens in normal and malignant human breast tissue Cancer Res 1985 45 2900 2906 3986816

[B25] Thijssen JHH Blankenstein MA Miller WR Milewicz A Estrogens in tissues: uptake from the peripheral circulation or local production Steroids 1987 50 297 306 10.1016/0039-128X(83)90079-X 3504065

[B26] Vermeulen A Deslypere JP Beck JS Biosynthesis of active oestrogens in the breast Oestrogen and the Human Breast 1989 Edinburgh: Royal Society of Edinburgh 195 201

[B27] Castagnetta L Granata O Blasi L Cassetti A Comito L Cavasino V Polito L Carruba G Recent studies on metabolism and concentrations of estrogens in breast cancer tissues and cells Breast Cancer Detect Prev 1992 16 S65-S70

[B28] Xu X Veenstra TD Fox SD Roman JM Issaq HJ Falk R Saavedra JE Keefer LK Ziegler RG Measuring fifteen endogenous estrogens simultaneously in human urine by high-performance liquid chromatography-mass spectrometry Anal Chem 2005 77 20 6646 6654 10.1021/ac050697c 16223252

[B29] Blonder J Johann DJ Veenstra TD Xiao Z Emmert-Buck MR Ziegler RG Rodriguez-Canales J Hanson JA Xu X Quantitation of steroid hormones in thin fresh frozen tissue sections Anal Chem 2008 80 22 8845 8852 10.1021/ac801402a 18937426 PMC7236062

[B30] Mueck AO Seeger H Lippert TH Estradiol metabolism and malignant disease Maturitas 2002 43 1 1 10 10.1016/S0378-5122(02)00141-X 12270576

[B31] Castagnetta LA Granata OM Traina A Ravazzolo B Amoroso M Miele M Bellavia V Agostara B Carruba G Tissue content of hydroxyestrogens in relation to survival of breast cancer patients Clin Cancer Res 2002 8 3146 3155 12374682

[B32] Rogan EG Badawi AF Devanesan PD Meza JL Edney JA West WW Higginbotham SM Cavalieri EL Relative imbalances in estrogen metabolism and conjugation in breast tissue of women with carcinoma: potential biomarkers of susceptibility to cancer Carcinogenesis 2003 24 697 702 10.1093/carcin/bgg004 12727798

[B33] Falk RT Xu X Keefer L Veenstra TD Ziegler RG A liquid chromatography-mass spectrometry method for the simultaneous measurement of 15 urinary estrogens and estrogen metabolites: assay reproducibility and interindividual variability Cancer Epidemiol Biomarkers Prev 2008 17 3411 8 10.1158/1055-9965.EPI-08-0355 19064556 PMC4158914

[B34] Im A Vogel VG Ahrendt G Lloyd S Ragin C Garte S Taioli E Urinary estrogen metabolites in women at high risk for breast cancer Carcinogenesis 2009 30 9 1532 5 10.1093/carcin/bgp139 19502596 PMC2736301

[B35] Gaikwad NW Yang L Muti P Meza JL Pruthi S Ingle JN Rogan EG Cavalieri EL The molecular etiology of breast cancer: evidence from biomarkers of risk Int J Cancer 2008 122 9 1949 57 10.1002/ijc.23329 18098283 PMC4422386

[B36] Hakkola J Pasanen M Pelkonen O Hukkanen J Evisalmi S Anttila S Rane A Mäntylä M Purkunen R Saarikoski S Tooming M Raunio H Expression of CYP1B1 in human adult and fetal tissues and differential inducibility of CYP1B1 and CYP1A1 by Ah receptor ligands in human placental and cultured cells Carcinogenesis 1997 18 391 397 10.1093/carcin/18.2.391 9054634

